# Urinary Tract Infections among HIV-Positive Pregnant Women in Mwanza City, Tanzania, Are High and Predicted by Low CD4+ Count

**DOI:** 10.1155/2017/4042686

**Published:** 2017-01-31

**Authors:** Tito Chaula, Jeremiah Seni, Nhandi Ng'walida, Alphaxaid Kajura, Mariam M. Mirambo, Rebekah DeVinney, Stephen E. Mshana

**Affiliations:** ^1^Department of Obstetrics and Gynecology, Catholic University of Health and Allied Sciences, P.O. Box 1464, Bugando, Mwanza, Tanzania; ^2^Department of Obstetrics and Gynecology, Bugando Medical Centre, P.O. Box 1370, Bugando, Mwanza, Tanzania; ^3^Department of Microbiology and Immunology, Catholic University of Health and Allied Sciences, P.O. Box 1464, Bugando, Mwanza, Tanzania; ^4^Department of Microbiology, Immunology and Infectious Diseases, Cumming School of Medicine, University of Calgary, 3330 Hospital Dr NW, Calgary, AB, Canada T2N 4N1

## Abstract

*Introduction*. Urinary tract infection (UTI) among pregnant women can lead to adverse maternal and foetal outcomes. UTI has been widely studied in the general obstetric population in Tanzania; the present study evaluated the magnitude, antimicrobial resistance, and predictors of UTI among HIV-positive pregnant women.* Methods*. Between March and May 2016 midstream urine samples from 234 women attending prevention of mother to child transmission of HIV (PMTCT) clinics were analyzed using standard methods. Data was analyzed by STATA version 11.0.* Results*. The prevalence of UTI was 21.4%, 50/234 [95% CI: 16.1–26.6]. The asymptomatically significant bacteriuria was higher than symptomatically significant bacteriuria (16.6% versus 4.7%, *p* < 0.001). On multivariable logistic regression analysis, single marital status (OR: 2.6, 95% CI: 1.1–6.1, and *p* = 0.026), low CD4+ counts of <200/*μ*L (OR: 2.9, 95% CI: 1.1–7.7, and *p* = 0.031), and having UTI symptoms (OR: 2.5, 95% CI: 1.1–6.0, and *p* = 0.03) were independent predictors of UTI.* Escherichia coli* predominated (57.7%) and exhibited a low prevalence of resistance to nitrofurantoin (16.7%), gentamicin (10.0%), and ceftriaxone (13.3%). Four (13.3%) of these were extended-spectrum beta-lactamase producers.* Conclusions*. A considerable proportion of HIV-positive pregnant women in Mwanza have significant bacteriuria which calls for the need to introduce routine UTI screening at PMTCT clinics to guide specific treatment and prevent associated complications.

## 1. Introduction

Urinary tract infection (UTI) is a common clinical condition among women, in particular pregnant women, due to anatomical and physiological factors [1–3]. Both symptomatic and asymptomatic UTI can negatively affect pregnant women and their foetuses [2–4]; moreover the increased likelihood of recurrence, even after successful treatment, complicates its management [5].

Human immunodeficiency virus (HIV) results in increased likelihood of opportunistic infections, including UTI, especially in developing countries where limited health services are available [1, 6]. Studies have shown higher prevalence of asymptomatic bacteriuria (ASB) among HIV-infected women than among uninfected pregnant women [7–9]. Also, the use of trimethoprim-sulfamethoxazole prophylaxis routinely in this population may potentially increase the risk of developing multidrug resistant (MDR) bacterial infections [9, 10].

In the northwestern part of Tanzania, a previous study conducted seven years ago revealed that 13.0 to 17.9% of pregnant women have UTI largely due to* Escherichia coli* and* Enterococcus* spp. [11]. Approximately 30% of the* E. coli* isolates were resistant to ceftriaxone [11]. However, it is not known whether CD4+ count level, initiation of HAART, and trimethoprim-sulfamethoxazole prophylaxis, as recommended by the Ministry of Health, Community Development, Gender, Elderly and Children (MOHCDGEC) guidelines, have an impact on the prevalence of UTI, bacterial etiology, and antimicrobial resistance profiles among HIV-infected pregnant women. The present study was therefore conducted to assess the epidemiological changes pertaining the magnitude of UTI, predictor variables, and antimicrobial resistance profiles among HIV-infected pregnant women.

## 2. Methods

### 2.1. Study Settings, Design, and Participants

This was a hospital-based, cross-sectional study conducted from March to May 2016 at PMTCT clinics at Bugando Medical Centre (BMC), Sekou Toure Regional Hospital, Nyamagana District Hospital, and Makongoro and Buzuruga Health Centres, all located in Mwanza City, northwestern Tanzania. All HIV-infected pregnant women attending PMTCT clinics in these health facilities during the study period and who consented to participate in the study were enrolled, whereas HIV-infected pregnant women known to have urinary tract abnormalities were excluded.

### 2.2. Sample Size and Sampling Procedures

The minimum sample size for this study was determined with the Kish Leslie formula (1965) using the prevalence of 18.1% among HIV-infected pregnant women having asymptomatic bacteriuria in Nigeria at 95% confidence interval [9]. A total of 234 HIV-infected pregnant women were enrolled.

### 2.3. Data Collection and Laboratory Procedures

A structured data collection tool was used to collect sociodemographic, obstetrics, and laboratory information. Patient CD4+ counts, HAART treatment, and other clinical information were extracted from the patients' files. For participants without recent (within 6 months) determination of CD4+ counts, blood samples were taken and processed using BD FACScallibur™ to obtain current CD4+ levels [12].

Participants were instructed on how to collect clean-catch midstream urine (MSU) and place it into screw-capped, wide-mouthed, sterile disposable plastic containers [13]. Urine samples were taken to the CUHAS multipurpose laboratory and processed within an hour, or in some cases they were placed in cold box at 4°C and processed within 4 hours.

Urine specimens were inoculated onto MacConkey Agar and Blood Agar plates (OXOID, Hampshire, United Kingdom) and incubated at 37°C for 18 to 24 hours. Diagnosis of UTI was made based on the presence of ≥10^5^ colony-forming-units per milliliter of MSU of one or two types of bacterial species. Samples with more than two types of bacteria species were regarded as contamination, and sample collection was repeated [13]. Identification of bacterial isolates was done by using biochemical identification tests as previously described [13].

Antimicrobial susceptibility testing of the isolates was done using the Kirby Bauer disc diffusion test on Muller Hinton agar (OXOID, Hampshire, United Kingdom) following the Clinical Laboratory Standard Institute (CLSI) guidelines [14]. Commonly used antimicrobial agents tested for Gram-positive bacteria were penicillin (10 units), erythromycin (15 *μ*g), trimethoprim-sulfamethoxazole (1.25/23.75 *μ*g), tetracycline (30 *μ*g), nitrofurantoin (300 *μ*g), ciprofloxacin (5 *μ*g), and vancomycin (30 *μ*g). For Gram-negative bacteria, antimicrobial agents tested were ampicillin (10 *μ*g), amoxicillin-clavulanate (20/10 *μ*g), piperacillin-tazobactam (100/10 *μ*g), trimethoprim-sulfamethoxazole (1.25/23.75 *μ*g), nitrofurantoin (300 *μ*g), gentamicin (10 *μ*g), ciprofloxacin (5 *μ*g), ceftriaxone (30 *μ*g), ceftazidime (30 *μ*g), and meropenem (10 *μ*g). Extended-spectrum beta-lactamase (ESBL) production was concomitantly tested in the same Muller Hinton agar plate using the double disk synergy method as previously described [14, 15].

### 2.4. Data Management

Using Excel data spread sheet, data were double entered and transferred to STATA version 11.0 for analysis. All categorical variables were summarized into proportions or frequencies, whereas continuous variables were summarized using means (±standard deviations) and median (interquartile range) depending on the distribution of data. *T*-test and Wilcoxon rank-sum tests were done to compare the difference between mean and median of different groups, respectively. Univariable, followed by multivariable, logistic regression analysis for the factors with a *p* value less than 0.2 was done to calculate odds ratio and 95% confidence interval. A *p* value of less than 0.05 was considered significant.

### 2.5. Data Quality Control

Data were double entered into the excel spread sheet to ensure accuracy and reliability. Laboratory procedures were performed by laboratory scientists under the supervision of clinical microbiologists to ensure quality results.* Escherichia coli* ATCC 259922 and* Staphylococcus aureus* ATCC25923 were used as reference strains for quality control of laboratory tests.

### 2.6. Ethical Considerations

The study was approved by the joint CUHAS/BMC Research Ethics and Review Committee (CREC113/2016). Permission was sought from the Departments of Obstetrics and Gynecology at BMC, Sekou Toure Regional Hospital, Nyamagana District Hospital, and Makongoro and Buzuruga Health Centres. Written informed consent was obtained from every participant before data and sample collection. For women aged below 18 years, consent was sought from the parent/guardian, and they were requested to assent to the study. Results for antimicrobial susceptibility testing were timely communicated to the attending doctors/nurses for management.

## 3. Results

### 3.1. Sociodemographic Characteristics of the Study Participants

A total of 234 HIV-infected pregnant women, aged between 17 and 43 years, were enrolled in the study; the mean age of study population was 28 ± 5.5 years. All pregnant women were from urban areas. The majority of women in the study were married 203 (86.8%) (Table 1).

### 3.2. Clinical and Obstetric Characteristics of HIV-Infected Pregnant Women

A total of 111 (47.4%) HIV-infected pregnant women were in the 3rd trimester. The median baseline and recent CD4+ counts were 264 (IQR 108–434) cells/*μ*L and 407.5 (291–594) cells/*μ*L, respectively. The median duration on HAART was 16 (5–34) months. Of the 234 participants, 215 (91.9%) were using trimethoprim-sulfamethoxazole prophylaxis daily (Table 2).

### 3.3. Prevalence of UTI and Bacterial Species Isolated

**Figure 1:**
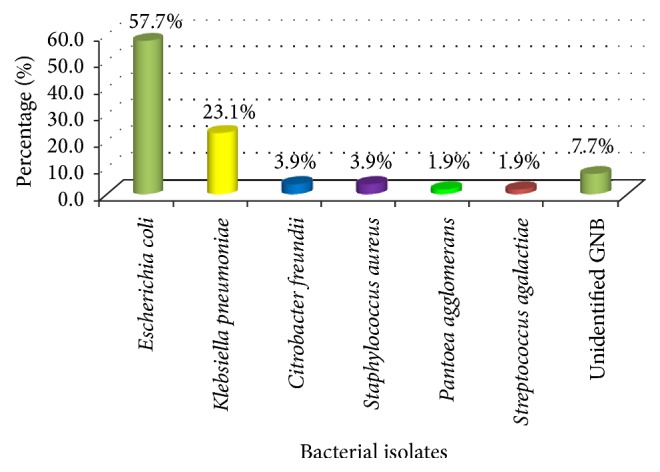
Bacterial species isolated from HIV-positive pregnant women with significant bacteriuria. Total number of bacteria is 52; two patients had dual bacterial infection; GNB: Gram-negative bacteria.

Of the 234 participants, 50 (21.4%, 95% CI: 16.1–26.6) had UTI. Out of 234 women, 39 (16.6%) had asymptomatically significant bacteriuria compared to 11 (4.7%) of women with symptomatically significant bacteriuria (*p* < 0.001).* E. coli* 30 (57.7%) and* Klebsiella pneumoniae* 12 (23.1%) were the most frequent bacterial species isolated (Figure 1).

### 3.4. Rate of Resistance to Antimicrobial Agents

Observed rates of resistance among* E. coli* were ampicillin 93.3%, trimethoprim-sulfamethoxazole 90.0%, nitrofurantoin 16.7%, gentamicin 10.0%, ceftriaxone 13.3%, and meropenem 3.3%. For* K. pneumoniae* isolates, the resistance rates were 100%, 72.7%, 33.3%, 0.0%, and 0.0% to ampicillin, trimethoprim-sulfamethoxazole, nitrofurantoin, ceftriaxone, and meropenem, respectively. The proportion of extended-spectrum beta-lactamase- (ESBL-) producing Gram-negative bacteria was 8.2% (4/49), all of which were* E. coli*, 13.3% (4/30) (Table 3).

Two* Staphylococcus aureus* isolates were resistant to penicillin, trimethoprim-sulfamethoxazole, tetracycline, and nitrofurantoin; all were sensitive to erythromycin, ciprofloxacin, gentamicin, and vancomycin. Furthermore, one* Streptococcus agalactiae* isolate was resistant to ampicillin, trimethoprim-sulfamethoxazole, nitrofurantoin, and erythromycin; it was sensitive to ciprofloxacin, gentamicin, and vancomycin.

### 3.5. Factors Associated with UTI among HIV-Infected Pregnant Women

Multivariable logistic regression analysis showed that being single (OR: 2.6, 95 CI: 1.1–6.1, and *p* = 0.026), current low CD4+ counts of <200/*μ*L (OR: 2.9, 95% CI: 1.1–7.7, and *p* = 0.031), and UTI symptoms (OR: 2.5, 95% CI: 0.6–8.3, and *p* = 0.03) were independent predictors of UTI among HIV-positive pregnant women. Coitus frequency was also associated with UTI on bivariate analysis (12.12% and 25.0% among those practicing coitus more than two and one times per week, resp.). Nevertheless, this variable was omitted in multivariate analysis due to its collinearity with marital status (Table 4).

## 4. Discussion

UTI is common among HIV-infected pregnant women, and, if left untreated, it can lead to poor maternal and foetal outcomes [4, 6]. The prevalence of UTI in this study was higher than approximately 15% observed in the general obstetric population in the same region measured seven years ago [11]. In the previous study, significantly higher rates in symptomatically significant bacteriuria was observed [11], while in the present study more women presented with asymptomatic bacteriuria. This connotes a rising trend of UTI, from approximately 15% in 2009 to 21% in the current study, with preponderance of UTI in the vulnerable HIV-infected pregnant women.

In this study,* E. coli* was the most common uropathogen isolated, a result that is comparable to the previous findings at BMC, Muhimbili, and Hydom in Tanzania among the general obstetric population [4, 11, 16]. The similarity of these results may be due to the predominance of* E. coli* in different populations in these settings [17–20] and the anatomical proximity between anus, vagina, and urethra in relation to the hygienic behavior.

The low level of resistance among uropathogens to nitrofurantoin, gentamicin, and ceftriaxone reiterates the fact that these antimicrobial agents may be judiciously used as potential therapeutic options in this population. Of note, when comparing the current findings with the previous study in the same setting [11], there has been a rise in resistance trends for trimethoprim-sulfamethoxazole (64.7% to 90.0%), nitrofurantoin (5.9% to 16.7%), and gentamicin (5.9% to 10.0%) among* E. coli* isolates. High levels of resistance to commonly used antimicrobial agents, such as ampicillin and trimethoprim-sulfamethoxazole, were also reported in Ghana and Ethiopia [21, 22]. These results emphasize the need to strengthen AMR control strategies in developing countries so that antimicrobial agents can be preserved for future generations. ESBL production was found in 13.3%; this is worrisome as women with these pathogens were outpatients and therefore represent community acquired UTI. One plausible explanation may be related to previous exposure to antimicrobial agents, which then selects drug-resistant mutants in the digestive tract. The mutant strains in turn contaminate the urinary system and cause reinfection. This emphasizes the need to strengthen antimicrobial stewardship and strategies that will change human behavior on issues related to antibiotic use, disposal, and improved hygienic measures.

Several studies have documented various predictors of UTI [8, 9]. In the present study, single marital status, current lower CD4+ count below 200 cells/*μ*L, and the presence of symptoms predicted UTI. Several other studies have shown no association of UTI and marital status [9, 11]. Although no clear explanation was revealed by the present study, this may be attributable to the differences in cultural practice, hygiene, and norms on sex issues in different areas [23].

Similar to other reports [7, 9, 24], low CD4+ counts in this study predicted UTI among HIV-infected pregnant women, an observation that could be explained by the severity of immunosuppression and the increased likelihood for opportunistic infections, including UTI. In light of this finding, integration of routine CD4+ count measurement and UTI screening should be a continuing program to ensure rational and prompt management of this population.

Also, the presence of UTI symptoms among HIV-infected pregnant women was an independent predictor of UTI. This is similar to study findings reported elsewhere [25]. Because both symptomatic and asymptomatic bacteriuria can have negative maternal and foetal outcomes, routine UTI screening is highly recommended.

The use of trimethoprim-sulfamethoxazole as prophylaxis was not associated with UTI in this study, similar to another study in our setting among nonpregnant, HIV-infected individuals [20]. In contrast, a study performed in Uganda found that trimethoprim-sulfamethoxazole use was protective against significant bacteriuria [24]. The difference could be explained by other factors, such as CD4 counts. However, the high resistance prevalence emphasizes the fact that trimethoprim-sulfamethoxazole may not work as a therapeutic option for HIV patients with UTI.

## 5. Conclusion

The prevalence of UTI among HIV-infected pregnant women is high and is predicted by low CD4+ count of <200 cells/*μ*L, being single, and exhibiting UTI symptoms. We observed a predominance of* E. coli* exhibiting high resistance to ampicillin and trimethoprim-sulfamethoxazole compared to lower values for nitrofurantoin, gentamicin, and ceftriaxone.

We recommend introduction of routine UTI screening at PMTCT clinics among pregnant women at booking to guide specific treatment and prevent associated complications. Low CD4+ count <200 cells/*μ*L, being single, and the presence of urinary tract infection symptoms can be used as predictors of UTI when planning for selective screening in areas where urine culturing is not routinely performed. Studies evaluating obstetric and neonatal outcomes among HIV-infected pregnant women with UTI, as well as molecular characterization of these strains in the context of infection prevention and control, would be of interest for the future studies.

## Figures and Tables

**Table 1:** Sociodemographic characteristics of HIV-infected pregnant women.

Patient characteristics	Frequency	Percentage (%)
*Mean age (year)* ^*∗*^	28 ± 5.5	—
*Marital status*		
Married	203	86.8
Single	19	8.1
Divorced	12	5.1
*Occupation *		
Peasant	26	11.1
Housewife	134	57.3
Petty trader	64	27.3
Employed	10	4.3
*Religion *		
Christian	177	75.6
Muslim	57	24.4
*Education level*		
None	7	3.0
Primary	176	75.2
Secondary	48	20.5
College	3	1.3

^*∗*^Continuous variable.

**Table 2:** Clinical and obstetric characteristics of HIV-infected pregnant women.

Patient characteristics	Frequency	Percentage (%)
*Gravidity*		
Gravida 1	35	14.9
Gravida 2	57	24.4
≥Gravida 3	142	60.7
*Parity *		
Nulliparity	43	18.4
Para 1–3	149	63.7
≥Para 4	42	17.9
*Gestation age*		
1st trimester	13	5.6
2nd trimester	110	47.0
3rd trimester	111	47.4
*Coitus per week*		
≤1	168	71.8
≥2	66	28.2

*Median baseline CD4* ^**∗**^	264 (IQR 108–434)	
*Baseline CD4*		
≤200	141	60.3
>200	93	39.7
*Median current CD4* ^**∗**^	407.5 (IQR 291–583)	
*Current CD4*		
<200	21	9.0
≥200	213	91.0
*Median HAART duration (months)* ^*∗*^	16 (IQR 5–34)	
*Duration on HAART (months)*		
≤12	105	44.9
13–24	41	17.5
≥25	88	37.6
*Trimethoprim-sulfamethoxazole use*		
Yes	215	91.9
No	19	8.1
*Urinary symptoms*		
Yes	27	11.5
No	207	88.5

^**∗**^Median with interquartile range; IQR: interquartile range; HAART: highly active antiretroviral therapy.

**Table 3:** Antimicrobial resistance profiles among Gram-negative bacteria isolates.

Bacteria	AMP *n* (%)	SXT *n* (%)	NF *n* (%)	CIP *n* (%)	GN *n* (%)	AMC *n* (%)	CRO *n* (%)	MEM *n* (%)	ESBL *n* (%)
*E. coli* (*N* = 30)	28 (93.3)	27 (90.0)	5 (16.7)	4 (13.3)	3 (10)	13 (43.3)	4 (13.3)	1 (3.3)	4 (13.3)
*K. pneumoniae* (*N* = 12)	11 (100)	8 (72.7)	4 (33.3)	1 (9.1)	0 (0)	6 (54.5)	0 (0.0)	0 (0)	0 (0.0)
*Other GNB* (*N* = 6)	6 (100)	5 (83.3)	0 (0)	0 (0)	2 (33.3)	2 (33.3)	1 (16.7)	0 (0)	0 (0)

AMP: ampicillin; SXT: trimethoprim-sulfamethoxazole; NF: nitrofurantoin; AMC: amoxicillin-clavulanate; CRO: ceftriaxone; MEM: meropenem; GNB: Gram-negative bacteria; other GNB: *Citrobacter freundii* (2), *Pantoea agglomerans* (1), and unidentified GNB (3).

**Table 4:** Univariable and multivariable logistic regression analysis of the factors associated with UTI among HIV-infected pregnant women.

Characteristics	UTI positivity *n* (%)	Univariable OR (95% CI )	*p* value	Multivariable OR (95% CI)	*p* value
*Age* ^*∗*^	50 (28.6 ± 5.6)	0.97 (0.91–1.02)	0.253		

*Marital status*					
Married (203)	38 (18.7)	1			
Single (31)	12 (38.7)	2.74 (1.22–6.13)	0.012	2.61 (1.12–6.09)	0.026

*Occupation*					
Peasant (26)	3 (11.5)	1			
Employed (74)	35 (26.1)	1.48 (0.38–5.73)	0.567	1.4 (0.35–5.7)	0.623
Housewife (134)	12 (16.2)	2.71 (0.77–9.59)	0.122	2.24 (0.61–8.2)	0.223

*Religion *					
Muslim (57)	12 (21.1)	1			
Christian (177)	38 (21.5)	1.15 (0.49–2.13)	0.947		

*Education level*					
Secondary (57)	10 (19.6)	1			
Primary (177)	40(21.9)	1.16 (0.53–2.49)	0.729		

*Gravidity* ^*∗∗*^	3 (IQR 2–4)	0.88 (0.73–1.07)	0.21		

*Gestation age* ^*∗∗*^	28 (IQR 20–32)	1.03 (0.98–1.07)	0.24		

*Coitus per week*					
≥2 (66)	8 (12.1)	1			
≤1 (168)	42 (25.0)	2.42 (1.07–5.47)	0.034		

*Baseline CD4*					
>200 (93)	25 (17.7)	1			
≤200 (141)	25 (26.9)	1.7 (0.908–3.20)	0.97		

*Current CD4*					
≥200 (213)	41 (19.3)	1			
<200 (21)	9 (42.9)	3.15 (1.24–7.97)	0.02	2.92 (1.10–7.71)	0.031

*Duration ART (months) 234* ^*∗∗*^	15.5 (IQR 6–30)	1.0 (0.99–1.02)	0.93		

*SXT use*					
No	45 (20.9)	1			
Yes	5 (26.3)	1.35 (0.46–3.94)	0.58		

*UTI symptoms *					
No 207	39 (18.8)	1			
Yes 27	11 (40.7)	2.99 (1.27–6.88)	0.01	2.52 (1.05–6.04)	0.03

^**∗**^Mean and standard deviation (SD), ^**∗∗**^median with interquartile range (IQR), and SXT: trimethoprim-sulfamethoxazole.

## Additional Points

*Study Limitations.* HIV viral load, which has been reported to be potential predictor for UTI, was not measured in this study due to financial constraints.
